# Trimethoprim-Sulfamethoxazole-Induced Drug Reaction With Eosinophilia and Systemic Symptoms (DRESS) Complicated by Acute Liver Failure

**DOI:** 10.7759/cureus.30852

**Published:** 2022-10-29

**Authors:** Naif Hindosh, Ragarupa Kotala, Kristi Nguyen, Alpiniano Pintor

**Affiliations:** 1 Internal Medicine, St. Luke's University Health Network, Easton, USA

**Keywords:** regiscar, trimethoprim-sulfamethoxazole (tmp-smx), drug-induced acute liver failure, sulfa drug, bactrim ds, drug reaction with eosinophilia and systemic symptoms (dress)

## Abstract

Drug reaction with eosinophilia and systemic symptoms (DRESS) is a delayed adverse drug reaction that is characterized by fever, cutaneous manifestation, enlarged lymph nodes, hematologic abnormalities, and organ involvement. Multiple medications have been reported to cause DRESS with the presentation varying from drug to drug. Some cases are mild and can be managed by stopping the causative agent along with supportive measures; however, other cases can lead to multi-organ failure requiring systemic corticosteroids and organ transplant. Acute liver failure is a rare manifestation of DRESS. We report a patient who had recently completed a course of trimethoprim-sulfamethoxazole and presented with low-grade fever, diffuse skin rash, eosinophilia, elevated liver enzymes, acute kidney injury, and thrombocytopenia. DRESS was subsequently diagnosed based on history, physical examination, and relatively negative workup for an alternate diagnosis. The patient eventually showed improvement with steroid therapy without the need for a liver transplant. Due to its pharmacogenetic susceptibility, it is essential to recommend avoiding the causative medication for the patient's family members.

## Introduction

Drug-induced liver injury (DILI) is a well-known disease that accounts for the most common etiologies of acute liver failure in the United States [1]. Reported symptoms with DILI include fatigue, low-grade fever, loss of appetite, nausea, vomiting, right upper quadrant pain, jaundice, pruritus, or dark urine. In advanced stages, patients may develop encephalopathy and coagulopathy suggesting progression to acute liver failure [2]. Most common medications that are involved in DILI are acetaminophen, anesthetics, non-steroidal anti-inflammatory drugs (NSAIDs), statins, herbals, and antimicrobial agents such as isoniazid, B-lactams, sulfonamides, in addition to antifungals and HIV antiretroviral therapy [3].

Drug reaction with eosinophilia and systemic symptoms (DRESS) is a serious delayed adverse drug reaction that manifests as severe cutaneous lesions and multi-organ failure with associated fever, hematologic abnormalities, and lymphadenopathy [4]. The clinical presentation usually appears two to six weeks from the time of exposure to the causative agent [5]. DRESS has a mortality rate of 10%, with most cases usually secondary to hepatic involvement with resultant fulminant hepatitis [6]. The most reported causative agents of this syndrome are antiepileptic drugs, allopurinol, sulfonamides, and antibiotics such as vancomycin, minocycline, ampicillin, and sulfamethoxazole-trimethoprim [7]. The best-used criteria for the diagnosis of this syndrome are based on the Registry of Severe Cutaneous Adverse Reactions (RegiSCAR) scoring system [7].

Trimethoprim-sulfamethoxazole (TMP-SMX), is a commonly used antimicrobial agent for prophylaxis and treatment purposes. It has a wide range of side effects including gastrointestinal symptoms, dermatological reactions, kidney injury, and hepatic toxicity [8]. 

We present a case of a 57-year-old male patient with acute liver failure in the setting of DRESS syndrome secondary to the recent use of TMP-SMX.

## Case presentation

A 57-year-old male of Asian descent presented with two weeks of complaints of intermittent fever, fatigue, and painless rash. He underwent surgical resection of inverted sinus papilloma and was prescribed a 14-day course of TMP-SMX six weeks before the presentation. He denied weight loss, night sweats, sore throat, cough, shortness of breath, chest pain, nausea, vomiting, diarrhea, abdominal pain, arthralgia, myalgia, hematuria, headache, and confusion. Vital signs were stable with the exception of a temperature of 100.4F. Physical exam was notable for icteric sclerae, jaundiced skin, and a diffuse purpuric rash, which was more prominent over all extremities with desquamation, without oral, ocular, or genital involvements. No lymphadenopathy was present in the cervical, axillary, or inguinal regions. Abdominal examination was benign with no hepatosplenomegaly. Neurological examination was completely normal. The patient denied recent travel, alcohol consumption, and illegal drug use. The initial laboratory workup is shown in Table 1.

**Table 1:** Laboratory workup results on admission. WBC: white blood count; BUN: blood urea nitrogen; eGFR: estimated glomerular filtration rate; AST: aspartate aminotransferase; ALT: alanine aminotransferase; ALP: alkaline phosphatase; INR: international normalized ratio; PT: prothrombin time; PTT: partial thromboplastin time.

Test	Value	Reference range
Hemoglobin	10.9	12-17 g/dL
WBC	9.56	4.31-10.16 Thousand/uL
Eosinophils %	29%	0-6%
Absolute eosinophils	2.77	0-0.4 Thousand/uL
Lymphocytes	11%	14-44%
Platelets	1	149-390 Thousand/uL
Sodium	136	136-145 mmol/L
Potassium	4.7	3.5-5.3 mmol/L
Chloride	102	100-108 mmol/L
BUN	20	5-25 mg/dL
Creatinine	2.51 (baseline 1)	0.6-1.3 mg/dL
Calcium	8.4	8.3-10.1 mg/dL
eGFR	27	
ALT	376	12-78 U/L
AST	283	5-45 U/L
ALP	1476	46-115 U/L
Total bilirubin	20.5	0.2-1 mg/dL
Direct bilirubin	15.68	0-0.2 mg/dL
Total protein	6.6	6.4-8.2 g/dL
Albumin	2.50	3.5-5 g/dL
INR	1.20	0.84-1.19
PT	15.2 seconds	11.6-14.5 seconds
PTT	38 seconds	17-27 seconds

The patient received platelet transfusion and was started on intravenous hydration and empiric intravenous broad-spectrum antibiotics. The infectious workup was negative for a tickborne disease, parasite infection, coronavirus disease 2019 (COVID-19), HIV, Ebstein-Barr virus (EBV), cytomegalovirus (CMV), hepatitis, A, B, and C infections. Two sets of blood cultures were negative as well. C-reactive protein and erythrocyte sedimentation rate (ESR) were slightly elevated; however, autoimmune workup including antinuclear antibody (ANA), anti-double-stranded DNA antibodies, antimitochondrial antibody (AMA), anti-smooth muscle antibody (ASMA), antineutrophil cytoplasmic antibodies (ANCA), creatine kinase, and C3 and C4 complements were all within normal range. Hematology workup was negative for hemolytic etiology, hemolytic uremic syndrome (HUS), thrombotic thrombocytopenic purpura (TTP), and disseminated intravascular coagulation (DIC). The peripheral smear results were unremarkable with no parasites noted on the parasite smear. Flow cytometry results were of no significance. Further testing showed normal acetaminophen, ceruloplasmin, and alfa antitrypsin levels with slightly elevated ferritin level. The urine drug screen was negative. Abdominal ultrasound and computed tomography scan were unremarkable. Liver Doppler ultrasound demonstrated patent hepatic vasculature. A liver biopsy was not performed due to severe thrombocytopenia. 

With no clear cause for the patient's presentation, the etiology was felt to be drug-induced due to TMP-SMX. Further history was obtained and the patient reported that his mother and brother had a history of severe reactions to TMP-SMX.

As infectious etiology was ruled out along with continuous worsening liver functions as shown in Table 2, antibiotics were stopped and intravenous methylprednisolone was started. The patient was then transferred to the liver transplant center. 

**Table 2:** Laboratory workup on hospital day 4. WBC: white blood count; eGFR: estimated glomerular filtration rate; AST: aspartate aminotransferase; ALT: alanine aminotransferase; ALP: alkaline phosphatase; INR: international normalized ratio; PT: prothrombin time; PTT: partial thromboplastin time.

Test	Value	Reference range
Hemoglobin	8.8	12-17 g/dL
WBC	16.6	4.31-10.16 Thousand/uL
Eosinophils %	33%	0-6%
Atypical lymphocytes	3%	0%
Platelets	13	149-390 Thousand/uL
Creatinine	1.24	0.60-1.3 mg/dL
eGFR	64	
ALT	3303	12-78 U/L
AST	955	5-45 U/L
ALP	1616	46-115 U/L
Total bilirubin	22.19	0.2-1 mg/dL
Direct bilirubin	17.56	0-0.2 mg/dL
Total protein	4.4	6.4-8.2 g/dL
Albumin	1.8	3.5-5 g/dL
INR	1.96	0.84-1.19
PT	22.1 seconds	11.6-14.5 seconds
PTT	33.3 seconds	17-37 seconds

While the patient was undergoing liver transplant evaluation, fortunately, his laboratory testing started to improve as shown in Table 3. He did not require the transplant eventually. The rash was completely resolved and he was discharged on a prednisone taper. 

**Table 3:** Laboratory workup results within two weeks of starting steroid therapy. WBC: white blood count; eGFR: estimated glomerular filtration rate; AST: aspartate aminotransferase; ALT: alanine aminotransferase; ALP: alkaline phosphatase; INR: international normalized ratio; PT: prothrombin time; PTT: partial thromboplastin time.

Test	Value	Reference range
Hemoglobin	10.3	12-17 g/dL
WBC	18	4.31-10.16 Thousand/uL
Eosinophils %	16%	0-6%
Atypical lymphocytes	0%	0%
Platelets	163	149-390 Thousand/uL
Creatinine	1.08	0.6-1.3 mg/dL
eGFR	60	
ALT	226	12-78 U/L
AST	56	5-45 U/L
ALP	598	46-115 U/L
Total bilirubin	11.8	0.2-1 mg/dL
Direct bilirubin	9.1	0-0.2 mg/dL
Total protein	4.8	6.4-8.2 g/dL
Albumin	1.8	3.5-5 g/dL
INR	1	0.84-1.19
PT	12.3 seconds	11.6-14.5 seconds

The patient continued to have close follow-ups with his primary care provider and hepatologist. His laboratory tests three months after discharge showed continuous improvement in liver functions as shown in Table 4.

**Table 4:** Laboratory tests three months after discharge from the hospital. WBC: white blood count; eGFR: estimated glomerular filtration rate; AST: aspartate aminotransferase; ALT: alanine aminotransferase; ALP: alkaline phosphatase; INR: international normalized ratio

Test	Value	Reference range
Hemoglobin	13.8	12-17 g/dL
WBC	6.95	4.31-10.16 Thousand/uL
Eosinophils %	0%	0-6%
Atypical lymphocytes	0%	0%
Platelets	186	149-390 Thousand/uL
Creatinine	1.12	0.60-1.30 mg/dL
eGFR	63	
ALT	99	12-78 U/L
AST	56	5-45 U/L
ALP	269	46-115 U/L
Total bilirubin	7.9	0.2-1 mg/dL
Direct bilirubin	4.1	0-0.2 mg/dL
INR	0.93	0.84-1.19

## Discussion

The incidence of DRESS is estimated to be one in every 1000-10000 exposures [9,10]. DRESS reaction is suggested to be mediated by T-cell hypersensitivity [11]. Antibiotics attribute to 74% of DRESS cases, and sulfonamides compromised 3% of the cases [9]. The sulfonamide component of TMP-SMX is the most common culprit of liver injury, which may manifest as cholestatic, hepatocellular, or mixed [12]. 

Patients who have slow acetylation are more prone to delayed hypersensitivity reactions. Two major metabolisms of sulfonamides reported are through N-acetylation and oxidation pathway by cytochrome P450. N-acetyltransferase levels invariably differed in slow acetylators, in most of the patients the enzyme was genetically determined. Slow acetylators utilize the cytochrome P450 pathway to metabolize sulfonamides. They generate more toxic metabolites such as hydroxylamines or nitroso causing cell death or more immunologic phenomena like DRESS. They are detoxified partially by conjugation with glutathione. The incidence of slow acetylators is 5-10% for Asian descendants [13,14].

DRESS was subsequently diagnosed based on history, physical examination, and relatively negative workup for alternate diagnoses such as viral infection, autoimmune disease, hemolytic etiology, or lymphoma. Other cutaneous adverse drug reactions such as Steven-Johnson syndrome (SJS), exanthematous drug reaction, or acute generalized exanthematous pustulosis (AGEP) were excluded based on the rash characteristics, degree of eosinophilia, the severity of visceral organ involvement, and the absence of oral, genital, and ocular involvement.

RegiSCAR scoring system has been utilized to help in confirming or excluding the diagnosis of DRESS [12]. Our patient had a score of 6, which highly suggested the diagnosis of DRESS. This is shown in Table 5.

**Table 5:** Registry of Severe Cutaneous Adverse Reactions (RegiSCAR) scoring system Total score:  <2: Excluded ; 2-3: Possible ; 4-5:Probable;  ≥6: Definite BSA: body surface area; HAV: hepatitis A virus; HBV: hepatitis B virus; HCV: hepatitis C virus; ANA: antinuclear antibodies

Item	-1	0	+1	Additional points	Our patient
Fever >101.3F	No	Yes			-1
Enlarged lymph node >1cm		No	Yes		0
Eosinophilia ≥0.7 × 10^9^		No	Yes	2 points if >1.5 x 10^9^	2
Atypical lymphocytes		No	Yes		1
Rash suggestive of DRESS: => facial edema, purpura, infiltration, desquamation.	No	Unknown	Yes		1
Extent ≥50% of BSA		No/Unkown	Yes		1
Skin biopsy suggestive of DRESS	No	Yes/Unknown			0
Organ involvement: 1 point for each organ involvement.		No	Yes	maximum score: 2	1
Disease duration ≥15 days	No/Unknown	Yes			0
Exclusion of other causes; add 1 point if 3 of the following tests are excluded: HAV, HBV, HCV, mycoplasma, chlamydia, ANA, blood culture.		No/Unknown	Yes		1
Final score					6

Treatment includes stopping the causative drug along with supportive therapy. Systemic glucocorticoids have been utilized in severe cases with organ involvement. Literature showed mixed outcomes in terms of using steroids in DRESS-induced liver injury. Two multicenter retrospective studies by Ichai et al. [15] and Lee et al. [16] concluded that glucocorticoid had no significance in improving DRESS-induced liver failure; however, other studies demonstrated better outcomes in similar cases when managed with extensive steroid therapy [12,17]. The study by Ichai et al. also demonstrated that seven out of 16 patients required liver transplants [15]. Our patient responded well to intravenous methylprednisolone therapy and prolonged prednisone taper. 

In terms of prognosis, complete recovery can take up to 12 weeks after stopping the offending drug [18,19]. It had been reported that patients who recover from DRESS, may develop a variety of autoimmune conditions [20,21 ]. CMV reactivation had been associated with the worst outcomes [22]. Even after full recovery, it has been shown that DRESS flare-up is a possibility and is more likely to be linked with patients who required systemic corticosteroid therapy during the acute event or had rapid prednisone taper [18,23].

## Conclusions

The presentation of DRESS is heterogenous and may lead to multiorgan failure. It is important to recommend avoiding the causative medications for the patient's family members as there is a link between several human leukocyte antigen (HLA) haplotypes and the probability of developing DRESS. Monitoring for developing autoimmune diseases is an essential part of follow-up management. Stopping the offending drug is the mainstay of treatment. Steroid therapy has been used in severe cases with organ involvement. Acute liver failure induced by DRESS can be reversible and liver transplant can be avoidable. 
